# Supplementary material for: Estimated Glomerular Filtration Rate as a Prognostic Factor in Urothelial Carcinoma of the Upper Urinary Tract: A Systematic Review and Meta-Analysis
Source: J Clin Med. 2021 Sep 15;10(18):4155. doi: 10.3390/jcm10184155 (PMC8472261; doi:10.3390/jcm10184155)
Supplement: Supplementary file 1 [file jcm-10-04155-s001.zip › jcm-1346790-supplementary.pdf]

Table S1. Assessment of quality of the non-randomized controlled trials (RCTs) using the Newcastle-Ottawa a scale for cohort studies.

Study	Selection				Comparability	Outcome			
	REC	SNEC	AE	DOI	CCB	AO	FLO	AFC	Score
Xylinas	★	★	★		★	★	★	★	7
Ito	★	★	★		★	★	★	★	7
Raman	★	★	★		★	★	★	★	7
Morizane	★	★	★		★	★	★	★	7
Yeh	★	★	★		★	★	★	★	7
Huang	★	★	★		★	★	★	★	7
Xing	★	★	★		★	★	★	★	7
Yu	★	★	★		★	★	★	★	7
Koguchi	★	★	★		★	★	★	★	7
Freifeld	★	★	★		★	★	★	★	7
Jan	★	★	★		★	★	★	★	7
Kuroda	★	★	★		★	★	★	★	7
Momota	★	★	★		★	★	★	★	7

REC: Representativeness of the exposed cohort; SNEC: Selection of the non-exposed cohort; AE: Ascertainment of exposure; DOI: Demonstration of outcome of interest; CCB: Comparability of cohorts based on design or analysis; AO: Assessment of outcome; FLO: follow-up long enough for outcomes to occur; AFC: Adequacy of follow-up of cohorts
